# Bizarre parosteal osteochondromatous proliferation (Nora’s lesion) affecting the distal end of the ulna: a case report

**DOI:** 10.1186/s12891-016-0981-3

**Published:** 2016-03-16

**Authors:** Yuichiro Matsui, Tadanao Funakoshi, Hideyuki Kobayashi, Tomoko Mitsuhashi, Tamotsu Kamishima, Norimasa Iwasaki

**Affiliations:** Department of Orthopaedic Surgery, Hokkaido University Graduate School of Medicine, Kita-15 Nishi-7, Kita-ku, Sapporo, 060-8638 Japan; Department of Surgical Pathology, Hokkaido University Hospital, Kita-14 Nishi-5, Kita-ku, Sapporo, 060-8648 Japan; Faculty of Health Sciences, Hokkaido University, Kita-12 Nishi-5, Kita-ku, Sapporo, 060-0812 Japan

**Keywords:** Bizarre parosteal osteochondromatous proliferation, Nora’s lesion, Distal end of ulna, Preoperative imaging studies, Bone decortication

## Abstract

**Background:**

Bizarre parosteal osteochondromatous proliferation (BPOP), first described by Nora et al. in 1983 and therefore termed “Nora’s lesion”, is a rare lesion that occurs in the short bones of the hands and feet and eventually presents as a parosteal mass. Reports of BPOP in the long bones are very rare. A benign disease, BPOP does not become malignant, although a high rate of recurrence following surgical resection is reported. Because of its atypical imaging findings and histopathological appearance, a BPOP might be misdiagnosed as a malignant tumor such as an osteochondroma with malignant transformation, a parosteal osteosarcoma, or a periosteal osteosarcoma.

**Case presentation:**

A 58-year-old woman complained of left ulnar wrist pain at the time of her initial presentation. Plain x-rays showed ectopic calcifications in and around the distal radioulnar joint, which supported the diagnosis of subacute arthritis with hydroxyapatite crystal deposition. She was initially given a wrist brace and directed to follow-up, but her persistent pain required the administration of corticosteroid injections into the distal radioulnar joint. Increasing ulnar wrist joint pain and limited forearm pronation and wrist flexion necessitated computed tomography and contrast-enhanced magnetic resonance imaging. BPOP was diagnosed based on the preoperative imaging studies, and a resection of the lesion was performed along with the decortication of the underlying the cortical bone to reduce recurrence rates. The diagnosis of BPOP was confirmed by pathologic examination. Two years after surgery, the patient has no subsequent pain complaints and an improved range of motion.

**Conclusions:**

BPOP affecting the distal end of the ulna is exceedingly rare. Because BPOP was diagnosed primarily based upon preoperative imaging findings in our patient, decortication of the underlying cortical bone was performed to reduce recurrence rates. Further careful follow-up in these patients is essential, despite the non-recurrence of the lesion.

## Background

Bizarre parosteal osteochondromatous proliferation (BPOP), first described by Nora et al. [1] in 1983 and therefore termed “Nora’s lesion”, is a relatively rare disease that most commonly presents as a parosteal mass in a short bone of the hands or feet. Reports of the lesion occurring in the long bones are very rare. BPOP is a benign disease and does not undergo malignant transformation, although a high rate of recurrence after surgical resection is reported [1–4]. Proliferation of the lesion occurs on the cortical surface, and it has no continuity with the normal medulla. Histologically, it is characterized by a heterogenous mixture of exophytic outgrowths from the cortical surface, which consist of bone, cartilage, and fibrous tissue [5]. To diagnose a BPOP it must be differentiated not only from an osteochondroma, but from malignant tumors such as osteochondroma with malignant transformation, parosteal osteosarcoma, and periosteal osteosarcoma. When surgery is needed, en bloc resection of the lesion followed by the decortication of the underlying cortical bone is important to reduce recurrence rates [6, 7].

We report here a case of BPOP at the distal end of the ulna, which was diagnosed based on findings from preoperative imaging studies.

## Case presentation

**Fig. 1:**
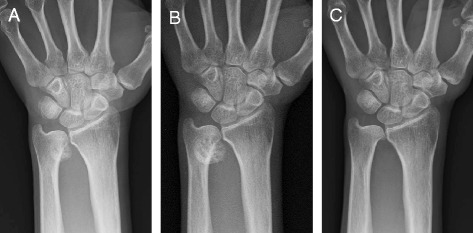
AP radiographic findings. **a** The first AP radiograph shows calcifications within the distal radioulnar joint (DRUJ). **b** The preoperative AP radiograph shows a bony prominence arising from the distal end of the ulna. **c** At follow-up 2 years after surgery. The AP radiograph indicates that there has been no recurrence of the lesion

**Fig. 2:**
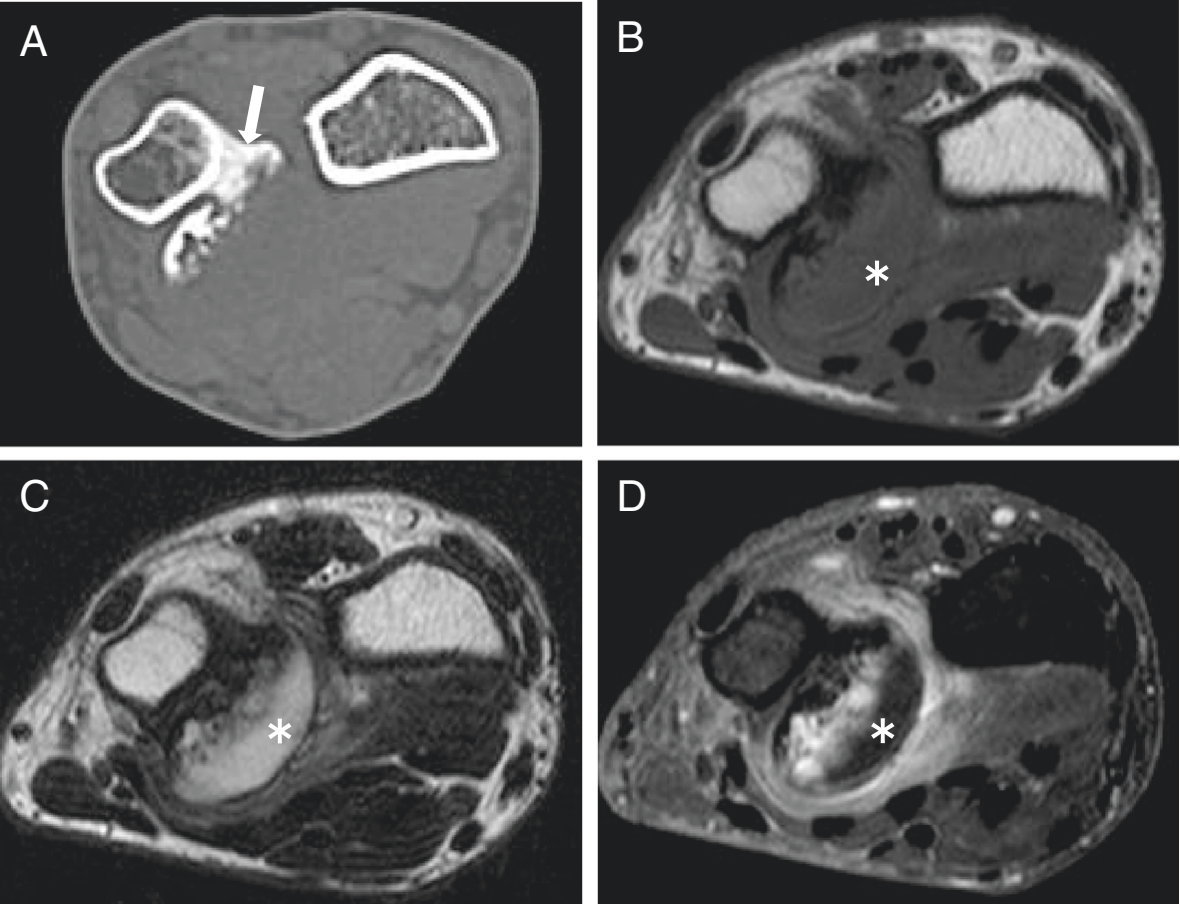
CT and MRI findings. **a** The CT scan shows a pedunculated bony prominence arising from the distal end of the ulna that has no continuity with the medullary cavity (arrow). **b** A axial T1-weighted MRI image that shows a low signal intensity at the margins of the lesion (asterisk). **c** A T2-weighted image that shows a high signal intensity at the margins of the lesion (asterisk). **d** A gadolinium-enhanced image shows a low signal intensity at the margins of the lesion (asterisk)

A healthy 58-year-old Japanese woman presented to a local clinic complaining of swelling and pain with motion of her left wrist joint, with no apparent trigger, beginning 3 months prior. Upon her initial presentation to our service, she complained of mild wrist pain without a limited range of motion. Plain x-rays showed ectopic calcifications in and around the distal radioulnar joint (DRUJ), and she was diagnosed as having subacute arthritis with hydroxyapatite crystal deposition (Fig. 1a). She was initially given a wrist brace and directed to follow-up, but her persistent pain required the administration of corticosteroid injections into the DRUJ. The treatment resulted in a transient improvement in her pain. However, she continued to experience a gradual worsening of her left ulnar wrist pain, and soon became aware of her limited range of motion. She therefore returned to our department 6 months after her initial presentation. At this time, an elastic, hard, non-mobile mass measuring 1 × 1 cm was palpated on the ulnar aspect of her left wrist. The range of motion of the wrist joint was 80°/65° on extension/flexion and 40°/80° on pronation/supination, demonstrating a limited range of motion of both flexion and pronation. Plain x-rays showed a bone mass at the distal end of the ulna (Fig. 1b), and computed tomography (CT) scans revealed a pedunculated bony prominence arising from the distal end of the ulna (Fig. 2a). On magnetic resonance imaging (MRI), the margins of the lesion had a low signal intensity on T1-weighted images (Fig. 2b), and a high signal intensity on T2-weighted images (Fig. 2c). An MRI with gadolinium contrast did not enhance the lesion (Fig. 2d). These MRI findings suggested that the margins of the lesion were consistent with proliferating cartilage. Based on our analysis of these images, we developed a differential diagnosis that included osteochondroma and BPOP. However, in the absence of continuity with the medulla, BPOP was primarily suspected. Moreover, MRI findings showed contrast enhancement of an area between the regions of proliferating cartilage and atypical bone formation detected by plain x-rays and CT scans. While this led to the inclusion of osteochondroma with malignant transformation, parosteal osteosarcoma, and periosteal osteosarcoma into the differential diagnosis, this intralesional contrast enhancement may also occasionally be seen in BPOP [3]. The preoperative imaging studies therefore led to the diagnosis of BPOP. We opted to perform surgery because of the patient’s worsening ulnar wrist pain and her limited range of motion.

**Fig. 3:**
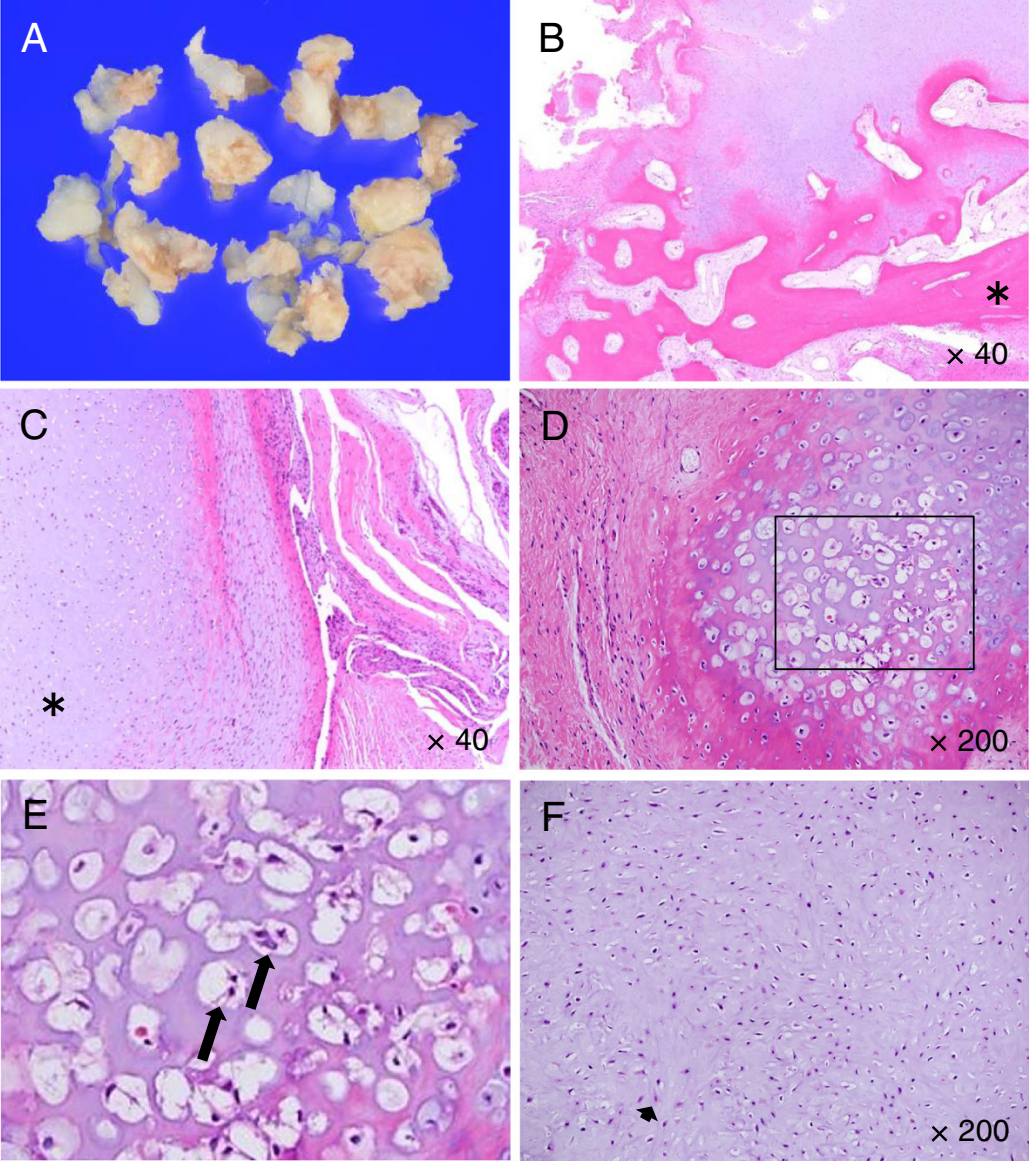
Gross and microscopic findings for the resected surgical specimen. **a** The gross photograph of the specimen shows that the surface of the lesion is covered by a cartilage cap and its interior is composed of osteoid tissue in continuity with the cortical bone. **b** Hematoxylin and eosin (H&E) staining of the tumor sections (magnification 40×). Cartilage was present at the margins of the lesion, and bone formation (asterisk) was found at the center of the lesion at its base fixed to the ulna. Bone trabeculae showed an irregular distribution, some of which was basophilic and incompletely ossified. There was also an area composed of a mixture of bone, cartilage, and fibrous granulation tissue that resembled a fracture callus. Fibrous vascular tissues were arranged loosely among the trabeculae, with little myeloid tissue. **c** H&E staining of the tumor sections (magnification 40×). The marginal cartilage (asterisk) was not the hyaline cartilage usually seen in the cartilage cap of osteochondromas, but resembled reactive fibrocartilage. **d** H&E staining of the tumor sections (magnification 200×). **e** Higher magnification views of the boxed areas of Fig. 3c. In some areas of the views with increased cellularity, star-shaped or spindle-shaped atypical cells were scattered in a somewhat myxomatous background (arrows). **f** H&E staining of the tumor sections (magnification 100×). Some of the chondrocytes exhibited mild atypia, such as nuclear enlargement and binucleation, without neoplastic osteoid production

The mass was identified through a volar approach. The surface of the lesion was covered by a cartilage cap and its interior was composed of osteoid tissue in continuity with the cortical bone (Fig. 3a). En bloc resection of the lesion with the capsule and the periosteum was performed along with the decortication of the underlying cortical bone to expose the underlying normal medulla. Histopathologically, cartilage was present at the margins of the lesion, and bone formation was found at the center of the lesion at its base fixed to the ulna (Fig. 3b). Because the marginal cartilage was not the hyaline type usually seen in the cartilage cap of osteochondromas but instead resembled reactive fibrocartilage (Fig. 3c), the possibility of an osteochondroma was ruled out. In some areas of the lesion with partially increased cellularity, star-shaped or spindle-shaped atypical cells (Fig. 3d and e) were scattered against a somewhat myxomatous background. Bone trabeculae showed an irregular distribution, some of which had a basophilic staining pattern (“blue bone”), representing incomplete endochondral ossification. There was also an area composed of a mixture of bone, cartilage, and fibrous granulation tissue that resembled fracture healing with callus formation. Fibrous vascular tissues were arranged loosely among the trabeculae, with little myeloid tissue. Some of the chondrocytes exhibited mild atypia, such as nuclear enlargement and binucleation (Fig. 3f). There was no neoplastic production of osteoid tissue. Therefore, the possibility of malignant tumors, such as a parosteal or periosteal osteosarcoma, was ruled out, and the diagnosis of BPOP was confirmed. Two years after surgery, the patient has no subsequent pain, an improved range of motion (80°/70° on extension/flexion and 80°/80° on pronation/supination), and no lesion recurrence (Fig. 1c).

## Discussion

BPOP is a relatively rare disease that most commonly presents as a parosteal mass in the short bones of the hands and feet. BPOP of the distal end of long bones is infrequent. BPOP has no gender preference [1]. It affects patients at any age, although most are in their 20s and 30s [4, 8]. Chromosomal anomalies associated with BPOP include t(1;17) (q32;q21) [9] and t(1;17) (q42;q23) [10]. BPOP expands broadly in continuity with the cortical bone, producing a mushroom-shaped mass. The lesion is often confused with an osteochondroma, as it is occasionally covered by a cartilage cap with no accompanying bone destruction. Because an osteochondroma occurs more often in the metaphyseal region of long bones, this association is particularly important when considering it as part of a larger differential diagnosis, as seen in this case. BPOP occasionally presents as calcification and ossification on X-ray images as seen in this patient. It may therefore be initially diagnosed and treated as calcinosis at an early stage. A thorough imaging study is therefore important to diagnosis.

On CT and MRI, BPOP lesions lack continuity with the medullary cavity, while osteochondromas maintain a continuity. The margins of the BPOP lesion in our case showed a low-signal intensity on T1-weighted images, and a high-signal intensity on T2-weighted images. The lesion was not enhanced on a MRI with gadolinium contrast, suggesting the proliferation of cartilage cells [3]. Because the lesion had no clear continuity with the medulla in this patient, BPOP was diagnosed based on preoperative MRI findings. Decortication of the underlying cortical bone was performed to reduce the tumor recurrence rate.

Histopathologically, cartilage was present at the margins of the lesion, irregular mature bone or evidence of ossification was found just beneath the bone trabeculae, and bone formation was found at the base of the lesion [4]. Because the marginal cartilage resembled reactive fibrocartilage, not the hyaline cartilage usually seen in the cartilage cap of osteochondromas, it was possible to distinguish the lesion from an osteochondroma. Additionally, the bone trabeculae were irregularly distributed, with some being basophilic and incompletely ossified. There was a mixed area of bone, cartilage, and fibrous granulation tissue resembling a fracture callus. Some of the chondrocytes had mildly atypical features, and there was no neoplastic production of osteoid tissue. Therefore, it was possible to differentiate BPOP lesions from malignant tumors, such as a parosteal or periosteal osteosarcoma.

BPOP has been reported to recur at high rates (20–55 %) after surgical resection [4, 11]. In general, observation alone is considered adequate for asymptomatic BPOP, but simple excision is indicated for patients with pain or a functional disorder. Resection of the capsule of the lesion and decortication of the underlying cortical bone is reportedly important to reduce recurrence rates [6, 7]. Because BPOP was diagnosed primarily based on preoperative MRI findings in our patient, such procedures should be performed. Thorough preoperative imaging studies should be considered essential to reach the correct diagnosis. Further long-term follow-up is warranted because recurrence is reported to occur from 10 to 120 months (49 months on average) after surgery [2].

## Conclusions

We reported a case of BPOP at the distal end of the ulna that was diagnosed based on findings from preoperative imaging studies. BPOP involving long bones like the distal end of the ulna is exceedingly rare. The local recurrence rate after surgical resection of the lesion is high. The preoperative diagnostic imaging study of our patient led to en bloc resection of the lesion along with decortication of the underlying cortical bone to reduce the possibility of recurrence. The final diagnosis was confirmed by the detailed histopathological findings. Two years after surgery, the patient has no subsequent pain and an improved range of motion. Further careful follow-up of such patients is essential even if there appears to be no recurrence of the lesion.

## Consent

Written informed consent was obtained from the patient for publication of this case report, along with any accompanying images. A copy of the written consent is available for review by the Editor of this journal.

## Abbreviations

BPOP: Bizarre parosteal osteochondromatous proliferation
CT: Computed tomography
DRUJ: Distal radioulnar joint
H&E: Hematoxylin and eosin
MRI: Magnetic resonance imaging

## References

[1] Nora FE, Dahlin DC, Beabout JW (1983). Bizarre parosteal osteochondromatous proliferations of the hands and feet. Am J Surg Pathol..

[2] Berber O, Dawson-Bowling S, Jalgaonkar A, Miles J, Pollock RC, Skinner JA (2011). Bizarre parosteal osteochondromatous proliferation of bone: clinical management of a series of 22 cases. J Bone Joint Surg Br..

[3] Dhondt E, Oudenhoven L, Khan S, Kroon HM, Hogendoorn PC, Nieborg A (2006). Nora’s lesion, a distinct radiological entity?. Skeletal Radiol..

[4] Meneses MF, Unni KK, Swee RG (1993). Bizarre parosteal osteochondromatous proliferation of bone (Nora’s lesion). Am J Surg Pathol..

[5] Gruber G, Giessauf C, Leithner A, Zacherl M, Clar H, Bodo K (2008). Bizarre parosteal osteochondromatous proliferation (Nora lesion): a report of 3 cases and a review of the literature. Can J Surg..

[6] Smith NC, Ellis AM, McCarthy S, McNaught P. Bizarre parosteal osteochondromatous proliferation: a review of seven cases. Aust N Z J Surg. 1996;66:694–7 10.1111/j.1445-2197.1996.tb00720.x 8855926

[7] Nilsson M, Domanski HA, Mertens F, Mandahl N (2004). Molecular cytogenetic characterization of recurrent translocation breakpoints in bizarre parosteal osteochondromatous proliferation (Nora’s lesion). Hum Pathol..

[8] Endo M, Hasegawa T, Tashiro T, Yamaguchi U, Morimoto Y, Nakatani F, et al. Bizarre parosteal osteochondromatous proliferation with a t(1;17) translocation. Virchows Arch. 2005;447:99–102. 10.1007/s00428-005-1266-7 15926071

[9] Campanacci DA, Guarracino R, Franchi A, Capanna R (1999). Bizarre parosteal osteochondromatous proliferation (Nora’s lesion). Description of six cases and a review of the literature. Chir Organi Mov.

[10] Michelsen H, Abramovici L, Steiner G, Posner MA (2004). Bizarre parosteal osteochondromatous proliferation (Nora’s lesion) in the hand. J Hand Surg Am..

[11] Joseph J, Ritchie D, MacDuff E, Mahendra A (2011). Bizarre parosteal osteochondromatous proliferation: a locally aggressive benign tumor. Clin Orthop Relat Res..

